# Acute and Late Toxicity after Moderate Hypofractionation with Simultaneous Integrated Boost (SIB) Radiation Therapy for Prostate Cancer. A Single Institution, Prospective Study

**DOI:** 10.1007/s12253-019-00623-2

**Published:** 2019-03-19

**Authors:** Kliton Jorgo, Csaba Polgar, Tibor Major, Gabor Stelczer, Andras Herein, Tamas Pocza, Laszlo Gesztesi, Peter Agoston

**Affiliations:** 1 Centre of Radiotherapy, National Institute of Oncology, Budapest Ráth György utca 7-9, Budapest, 1122 Hungary; 2 Department of Oncology, Semmelweis University, Budapest Üllői út 26, Budapest, 1085 Hungary; 3 Institute of Nuclear Technique, Budapest University of Technology and Economy, Budapest Műegyetem rakpart 3, Budapest, 1111 Hungary

**Keywords:** Prostate cancer, Simultaneous integrated boost, Moderate hypofractionation, Intensity-modulated radiotherapy, Image-guided radiotherapy

## Abstract

To evaluate the acute and late toxicity using moderately hypofractionated, intensity-modulated radiotherapy (IMRT) with a simultaneous integrated boost (SIB) to prostate for patients with intermediate and high risk prostate cancer. From 2015 to 2017, 162 patients were treated with IMRT with SIB to the prostate. IMRT plans were designed to deliver 50.4Gy in 28 fractions (1.8 Gy/fraction) to the pelvic lymph nodes (whole pelvis radiotherapy, WPRT) while simultaneously delivering 57.4 Gy in 28 fractions (2.05 Gy/fraction) to the seminal vesicles and 70 Gy in 28 fractions (2.5 Gy/fraction) to the prostate for high risk patients. For intermediate risk patients the same technique was applied, without WPRT. Acute and cumulative late genitourinary (GU) and gastrointestinal (GI) toxicities were scored according to the Radiation Therapy Oncology Group (RTOG) scoring system. Of the 162 patients enrolled, 156 (96%) completed the treatment as planned. The median follow-up time was 30 months. Seventy-eight patients (48.2%) were treated with WPRT. The rate of acute grade ≥ 2 GI and GU toxicities in all patients were 22% and 58%, respectively. The rate of cumulative late grade ≥ 2 GI and GU toxicities were 11% and 17%, respectively. Acute grade 3 GI and GU toxicities occurred in 1% and 1%. Late grade 3 GI and GU side effects occurred in 5% and 4%, respectively. None of the patients developed grade ≥ 4 toxicity. IMRT with SIB technique using moderate hypofractionation to the prostate is feasible treatment option for intermediate and high risk patients, associated with low rate of severe GU and GI toxicities.

## Introduction

Several randomized trials have shown improved biochemical control with dose escalation for prostate cancer [1–3]. The α/β ratio for prostate cancer is suggested to be lower than that of surrounding normal tissues, and may be as low as 1.5Gy [4, 5]. According to this, the therapeutic outcome using external beam radiotherapy is expected to be improved with hypofractionation, in case of delivering higher biologically effective dose (BED) than with conventional external beam radiotherapy. Asα/β ratio for rectum and urinary bladder is estimated to be 3Gy and 5-10Gy, respectively, the low α/β ratio ofprostate cancer theoretically allows dose escalation with hypofractionation without increasing late toxicity [6]. Beyond the advantages in terms of tumour control and late toxicity, the use of large dose per fraction is preferred by patients and may have important implications for cost-effectiveness by shortening the overall treatment time. Several contemporary phase III, randomized trials with mature data have confirmed similar tumour control and late toxicity among various hypofractionated regimens to conventionally fractionated external beam radiotherapy [7]. Independently of the patients risk groups, except one trial, the treatment target volume was the prostate gland and ± seminal vesicles without pelvic lymph nodes. The rational of elective whole pelvis radiotherapy (WPRT) in patients with high risk of subclinical lymph node involvement (Roach equation ≥15%) is the possibility to improve loco-regional control [8]. In these high risk patients, surgical series with extended lymphadenectomy showed a considerable incidence (17–46%) of microscopic disease in the pelvic lymph nodes [9–13]. Large, contemporary retrospective trials confirmed a statistically significant benefit in biochemical control with WPRT compared to prostate (± irradiation of seminal vesicles) irradiation, only [14–19]. However, currently there is insufficient evidence to recommend routine use of WPRT for high risk patients. Three randomized trials have shown no benefits for WPRT with respect to disease-free survival or overall survival [20–22]. Since 2015 we have treated intermediate and high risk prostate cancer patients with image-guided, intensity modulated radiotherapy (IG-IMRT), using a simultaneous integrated boost (SIB) to the prostate for moderate hypofractionation. The aim of this phase II study was to evaluate the acute and late side effects after moderate hypofractionation with or without WPRT in combination with SIB technique to the prostate.

## Materials and Methods

This prospective study was approved by our institutional ethical review board (68.2019). Patients with less than 12 months of follow up were excluded from the analysis. The main characteristics of the patients are shown in Table 1. Between January 2015 and March 2017, 162 patients with histologically confirmed adenocarcinoma of the prostate were enrolled in this trial. Before treatment a detailed medical history was recorded and physical examination including digital rectal examination was performed. A pretreatment bone scan and abdominal CT or MRI were required to stage the prostate cancer and to exclude distant metastases. Patients with prior pelvic irradiation, a history of collagen vascular or inflammatory bowel disease were also excluded. In accordance with D’Amico et al. [23], four risk groups were identified: low risk: ≤cT2a and Gleason score ≤ 6 andPSA <10 ng/mL; intermediate risk: cT2b and/or Gleason score 7and/or PSA 10–20 ng/ml; high risk: ≥cT2c and/or Gleason score ≥ 8 and/or PSA > 20 ng/mL and/or patients with cN1 disease. All patients participating in this study were intermediate or high risk or node positive. High risk and node positive patients received whole pelvic irradiation. According to our institutional protocol in high risk patients older than 70 years, considering the toxicity/benefit ratio, prophylactic WPRT was not performed, in the lack of high level of evidence proving better biochemical control or disease free survival after WPRT. Intermediate risk patients with bulky disease or unfavourable histological parameters received neoadjuvant and concomitant hormone therapy for 6 months (short-term hormone therapy). In high risk patients neoadjuvant-concomitant-adjuvant hormone therapy was given for 24–36 months (long-term hormone therapy). For patient immobilization supine or prone position is recommended [24]. In this study planning computed tomography (CT) imaging was performed in supine position using knee and ankle fixation support system for immobilization of the legs. Patients arms laid on their chest. Axial images were obtained with 3–5 mm slice thickness from L1 vertebra to about 3 cm below the ischial tuberosities. Before planning CT, patients were instructed to have moderately,comfortablyfilled bladder by drinking 0.5 l of water (after having it emptied) half an hour prior to CT and an emptyrectum. In case of habitual constipation light laxative was recommended. Four tattoos were marked on the skin at the time of planning CT.

**Table 1:** Patient, tumour and treatment characteristics

Characteristic	N (%)
Age (years)	
Median	71
Range	50–83
TURP^1^ before EBRT^2^	19 (11.,7%)
T stage	
T1–2	110 (68%)
T3	48 (30%)
T4	4 (2%)
N stage	
N0	140 (86%)
N1	22 (14%)
Gleason score	
≤6	33 (20%)
7	61 (38%)
≥8	68 (42%)
Initial PSA	
Median	18
Range)	2–400
<10	49 (30%)
10–20	39 (24%)
≥20	74 (46%)
Risk groups	
Intermediate	34 (21%)
High or lymph node positive	128 (79%)
Hormonal therapy	
No	16 (10%)
Short (≤ 6 months)	24 (15%)
Long (> 6 months)	122 (75%)

^1^TURP: transurethral resection of the prostate; ^2^EBRT: external beam radiotherapy

The rectum, bladder and hip joints were contoured as organs at risk (OARs). For intermediate risk 2 clinical target volumes were defined. The prostate clinical target volume (CTV_pros) included the whole prostate gland. The prostate and seminal vesicles CTV (CTV_psv) was generated by 5 mm expansion of CTV_pros in all directions except posteriorly at the prostate-rectum interface + proximal 1 cm of the seminal vesicles.

For high risk patients CTV_pros was the same as above. CTV_psv was defined by 5 mm expansion of CTV_pros in all directions except posteriorly + proximal 2 cm of seminal vesicles (in case of cT3b the entire seminal vesicles were included). Whole pelvis clinical target volume (CTV_pelv) consisted of CTV_psv + common iliac (under L5-S1 space), external iliac, presacral and obturator lymph nodes [25]. CTV_psv margins around CTV_pros were defined according to Chao et al. [26]. After analyzing a large number of prostatectomy specimens they found ≥4 mm extra capsular extension in 13% and 19% of the specimens in intermediate and high risk disease respectively.

Image guidance was performed using fiducial markers when pelvic irradiation was not required and with mega- or kilovoltage cone beam CT (MV or KV CBCT) in case of WPRT. During the whole treatment course daily orthogonal kilovoltage portal images were performed and fiducial markers were used for verification and online correctionof patients’ setup. In the WPRT group or in the cases of lacking fiducials CBCT and online correctionof patients’ setup was performed before the first 3 fractions. After the systematic error was calculated, isocentre was modified to exclude the systematic error, and thereafter CBCT based IGRT was used weekly for the rest of treatment. According to our institutional analysis, PTV margins with the use of fiducial and CBCT were 5 mm and 8 mm in every direction respectively.

All patients were treatedin 28 fractions with “step and shoot” IMRT or Volumetric Modulated Arc Therapy (VMAT) using 6–10 MV photon beams from Artiste (Siemens Medical Solutions Inc., USA) or TrueBeam and VitalBeam (Varian Medical Systems, Palo Alto, USA) linear accelerators, respectively.

**Fig. 1:**
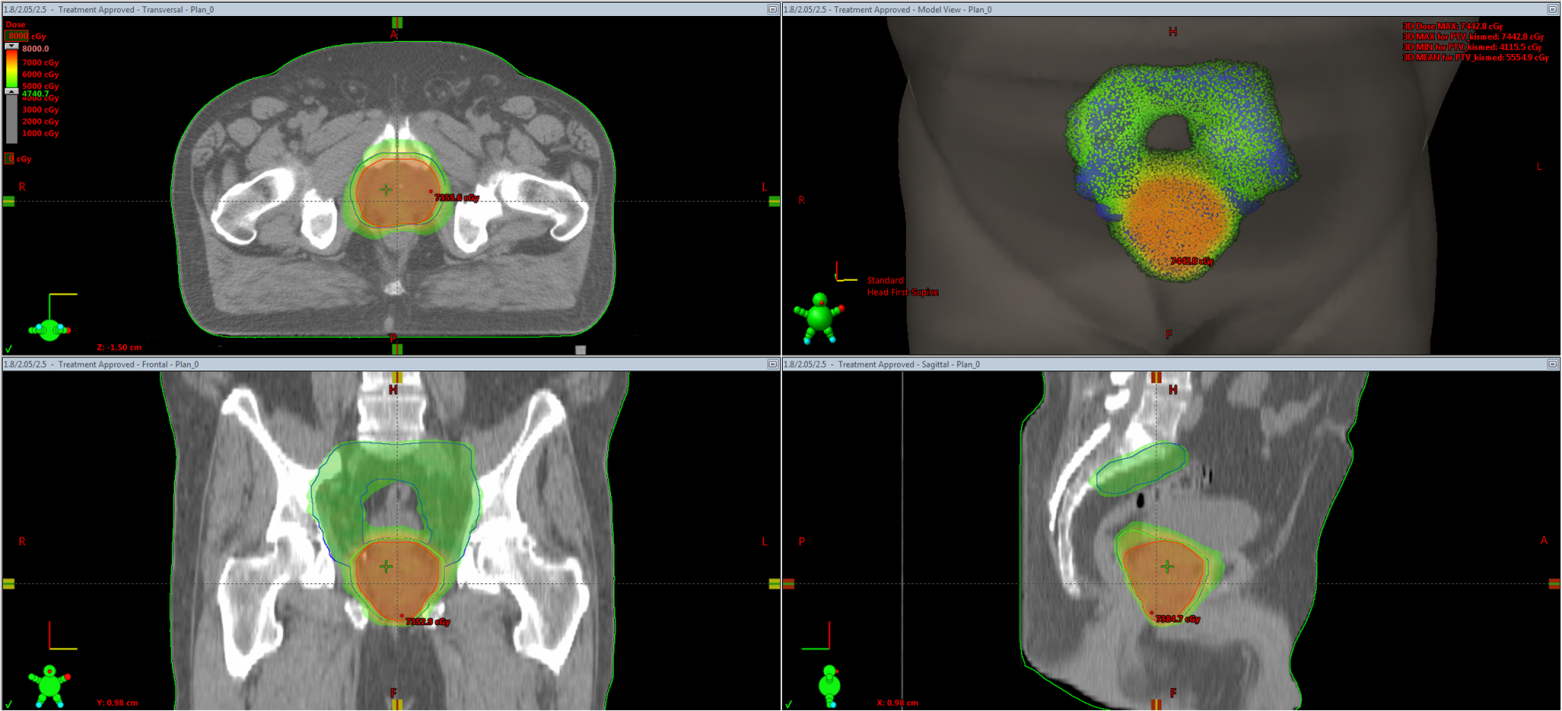
Representative dose distributions of a VMAT plan for high risk prostate cancer patient with whole pelvis irradiation in axial, sagittal, coronal and 3D views

Intermediated risk patients received 70 Gy to PTV_pros in 2.5Gy/fraction (EQD2 = 80 Gy) and 57.4 Gyto PTV_psv in 2.05 Gy/fraction (EQD2 = 58.2 Gy). High risk and lymph node positive patients received 70 Gy to PTV_pros, 57.4Gy to PTV_psv and 50.4 Gy to the pelvic lymph nodesusing 1.8 Gy/fraction. For PTV coverage, the 95% of the prescribed dose was requested to cover 95% of the target volume (V_95%_ > 95%) for all PTVs. Rectum V_45Gy_, V_63Gy_, bladder V_45Gy_, hip joints V_45Gy_was constrained below 50%, 20%, 65% and 10% of their volumes respectively. Dose constraint was not used regarding the small bowels. In Fig. 1 dose distribution is shown for the three target volumes in a high risk prostate cancer patient.

Patients were evaluatedevery second week during the treatment, every three months after radiation therapy for the first year and every 6 months thereafter. Acute and late genitourinary (GU) and gastrointestinal (GI) toxicities were scored according to the Radiation Therapy Oncology Group (RTOG) scoring system. Acute toxicities were reported during radiation therapy or within the initial 3-month follow-up.

Biochemical relapse free survival (bRFS) and cancer specific survival (CSS) curves were calculated using the Kaplan-Meier method. Spearman rank order and Chi-squared tests were used to evaluate the correlations between pelvic irradiation, method of image guidance, transurethral resection of prostate (TURP), hormone therapy, different dosimetric parameters of rectum, bladder, hip joints and acute GI, GU toxicities. Kaplan-Meier method and Gehan-Wilcoxon tests were used to evaluate the effects of pelvic irradiation, method of image guidance, TURP, hormone therapy on toxicity free survival. Logistic linear and Cox regressions were used in multivariate analysis to evaluate the prognostic factors of acute and late toxicities.

## Results

Of 162 patients 156 (96%) completed the moderately hypofractionated radiotherapy with SIB technique as planned. Seventy-eight patients (48.2%) were treated with WPRT, 84 patients (51,8%) with no WPRT. Six patients due to their age (74-82 year) and acute GU or GI grade 2 side effects received only 27 fractions (67.5 Gy to the prostate in 2.5 Gy fractions). Median follow-up was 30 months (range: 21–45).

The dosimetric data of the treatment plans are detailed in Table 2.Our dose constrains for rectum and hip joints have been reached in every patient. In 7 patients (4.3%) bladder V_45_ was >65% because of their relatively empty bladder at time of planning CT. Due to pre-treatment urinary symptoms these patients could not achieve filled bladder. IGRT was performed with fiducial markers, kV CBCT or MV CBCT in 32.7%, 61.1% and 6.2% of the patients, respectively.“Step and shoot” IMRT and VMAT techniques were applied in 6.2% and 93.8% of patients, respectively.

**Table 2:** Dose constraints and dosimetric parameters of organs at risk

Dosimetric parameter	Dose constraint (%)	Mean % (range)
Rectum V_45Gy_	50	29.8 (11–49)
Rectum V_63Gy_	20	9.5 (2–19)
Bladder V_45Gy_	65	36.4 (0.1–80)
Hip joints V_45Gy_	10	1.3 (0.1–9)

Acute and late toxicities are reported in Table 3.In general, urinary toxicity resolved spontaneously or with the administration of non-steroidal anti-inflammatory or α1 blockermedications. Six patients (4%) presented grade 3 late GU toxicity and 8patients (5%) grade 3 late GI toxicity. None of the patients experienced acute or late grade 4 side effects.

**Table 3:** Acute and cumulative late toxicities

Toxicity	Grade	Acute toxicity (%) *N* = 162	Late toxicity (%) N = 162
Gastrointestinal	0	27	83
	1	51	6
	2	21	6
	3	1	5
Genitourinary	0	11	68
	1	31	15
	2	57	13
	3	1	4

We found no correlation between acute or late toxicity andthe use of WPRT, previous urological surgery or modality of IGRT.

**Fig. 2:**
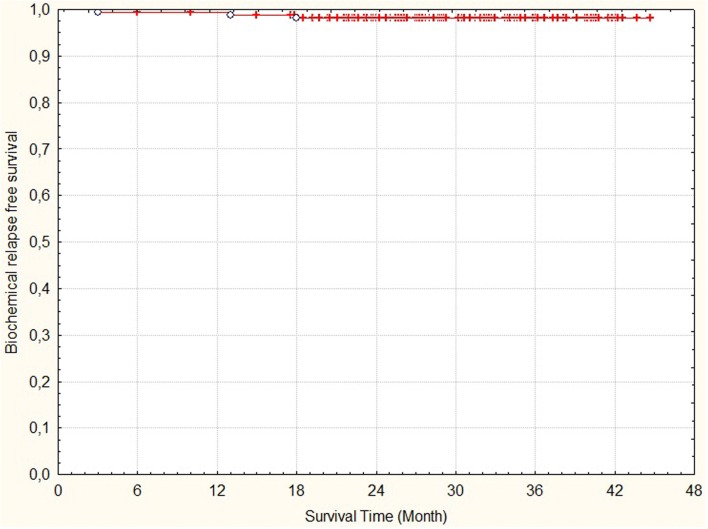
Time to biochemical failure

**Fig. 3:**
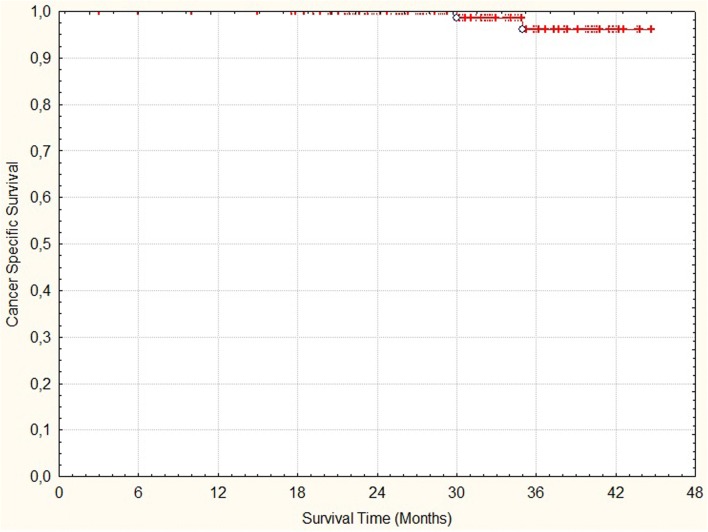
Time to prostate cancer death

Crude rate of biochemicalrelapse free survival and cancer specific survival was 98% and 99%, respectively (Figs. 2 and 3). No in-field lymph node recurrence was observed. Three patients (2%) developed multiplex bone metastases, currently one of them is receiving chemotherapy, 2 patients (1%) died due to disease progression. Six (4%) patients died from non-prostate cancer disease.

No correlation was found between WPRT vs. no WPRT, method of image guidance, TURP vs. no TURP, length of hormone therapy or the different dosimetric parameters of rectum, bladder, hip joints and acute GI, GU toxicities. Significant correlation was found between acute GI and acute GU toxicities, between acute GI and cumulative late GI and between acute GU and cumulative late GU toxicities (*p* < 0.05).

With Gehan-Wilcoxon test only a non-significant trend was observed for late GI toxicities in favour of image guidance with gold markers compared to CBCT based methods. For other parameters significant effects on toxicity were not found. In multivariate analysis no significant predictor was found for acute or late GI or GU toxicities.

## Discussion

Based on the radiobiological assumption that prostate cancer has a high sensitivity to fraction dose, due to a low α/β ratio (1.5Gy), several randomized trials were published, using a superiority (Regina Elena, Fox Chase, MD Anderson, HYPRO) and non-inferiority (NRG Oncology, CHHiP, PROFIT) design to compare moderate hypofractionation (dose per fraction 2.4–3.5 Gy) to conventional fractionation (dose per fraction 1.8–2 Gy) [27–35]. Both in superiority and non-inferiority trials the rate of late toxicity was similar with moderate hypofractionation compared to conventional fractionation provided that the dose per fraction was kept below 3 Gy. However, only in one of these trials (Fox Chase trial) the high risk patients were treated with WPRT. In this superiority randomized trial 303 patients received either 76 Gy in 2 Gy/fraction over 7.5 weeks or 70.2 Gy in 2.7 Gy/fraction while the pelvis was treated up to 56 Gy in 38 fractions in the conventional arm and up to 50 Gy in 26 fractions in the hypofractionated group. GI grade 3 toxicities occurred in 2% in both treatment arms, while GU grade 3 toxicities were observed in 3.3% and in 4%, respectively.

In high risk prostate cancer there is still considerable controversy in the literature regarding the role of elective pelvic node irradiation. In these patients there is a high probability of occult, radiologically undetectable lymph node metastases [8]. Based on the results of a randomized, phase III study comparing prostate only radiation therapy with WPRT, a trend to increased progression free survival was demonstrated in the group of patients with >15% probability of lymph node metastasis treated with WPRT [36, 37]. Another open issue regardingWPRT is the potential increase of toxicity, because of the larger irradiated volume compared to prostate only radiotherapy. Recently, several trials reported reduction of GI side effects using IMRT for irradiation of the pelvic lymph nodes [37, 38]. In the current trial none of the patient experienced grade 4 toxicity. Of 162 patients 2 (1%) developed acute GI and GU grade 3 side effects, late GI and GU grade 3 toxicities occurred in 8 (5%) and 6 (4%) patients, respectively.

Di Muzio et al. [39, 40] recently published their 5-year results of moderately hypofractionated radiation therapy with SIB to the prostate. Intermediate and high risk patients received 51.8Gy to pelvic nodes and concomitant SIB to the prostate up to 74.2 Gy in 28 fractions. Low risk patients were treated to the prostate only with 71.4Gy in 28 fractions. Among 211 patients the incidence of acute GU grade 2 and grade 3 toxicity was 29% and 1.9%, respectively, GI grade 2 and grade 3 toxicity occurred in 6.2% and 0.5%, respectively. Late GU grade ≥2 and grade ≥ 3 toxicity was 20.2% and 5.9%, late GI grade ≥2 and grade ≥ 3 toxicity 17% and 6.3% respectively. One patient experienced grade 4 toxicity (cystectomy).

Saracino et al. [41] reported 5-year results in 110 high risk patients treated with WPRT and SIB to the prostate. The 3- and 5-year rate of grade ≥ 2 late GI toxicity was 2% and 5%, respectively, while the 3- and 5-year rate of grade ≥ 2 late GU toxicity was 5% and 12%, respectively. The rate of grade 2 acute rectal, intestinal and genitourinary toxicities were 40%, 23%, and 39%, respectively, and none of the patients experienced grade 3 toxicity [42].

Franzese et al. [43] reported the results of 90 high risk patients treated with moderately hypofractionated radiotherapy with SIB. At 25 months late grade 2 and 3 GI toxicitywas observed in 1% and 0%, respectively. The rates of developing any late GU grade 2 or 3 toxicity were 6% and 1%, respectively.

The results of other important trials using WPRT combined with SIB to the prostate areshown in Table 4.

**Table 4:** Results of clinical trials using whole pelvis radiation therapy (WPRT) combined with simultaneous integrated boost (SIB) to the prostate. SV: seminal vesicles

Reference	No. patients	Total dose/fraction dose (Gy)	No. fractions	Median follow-up (months)	Acute toxicity grade ≥ 3 (%)		Late toxicity grade ≥ 3 (%)		Biochemical control (%)
					GU	GI	GU	GI	
McCammon, 2008 [44]	30	pelvis: 50.4/1,8	28	24	0	3.3	0	10	–
		prostate: 70/2.5							
Engels, 2009 [45]	28	pelvis: 54/1.8	30	10	4	0	–	–	–
		prostate: 70.5/2.35							
Adkison, 2010 [46]	53	pelvis: 56/2	28	25	0	0	2	0	81
		prostate: 70/2.5							
Alongi, 2012 [47]	70	pelvis: 51.8/1.85	28	11	1	0	–	–	–
		prostate: 74.2/2.65							
Pollack, 2013 [28]	303	pelvis: 50/1.92	26	68	–	–	4	2	76.7
		prostate: 70.2/2.7							
Saracino, 2014 [41]	37	pelvis: 57/1.56	40	56	0	0	0	0	90
		prostate: 80/2							
Di Muzio, 2016 [40]	211	pelvis: 51.8/1.85	28	60	1.9	0.5	5.9	6.3	95
		prostate: 74.2/2.65							
Franzese, 2017 [43]	90	pelvis: 51.8/1.8	28	25	2	0	1	0	90
		SV: 65.5/2.34							
		prostate: 74.2/2.65							
Chang, 2017 [48]	55	pelvis: 50.4/1.68	30	24	–	–	6	2	96
		SV: 54/1.8							
		prostate: 72/2.4							
Magli, 2017 [49]	41	pelvis: 50/2	25	60	0	0	0	0	95
		SV: 56.25/2.25							
		prostate: 67.5/2.7							
Present study	162	pelvis: 50.4/1.8	28	30	1	1	4	5	98
		SV: 57.4/2.05							
		prostate: 70/2.5							

In our prospective study with large number of patients, the toxicity rates compare favourably with similar trials using WPRT with SIB to the prostate. Despite the fact that in WPRT group the irradiated volume was larger compared to no WPRT group, no correlation was found between WPRT vs. no WPRT and acute GI, GU toxicities. To our opinion, the improved dose distribution of IMRT plans allowing a reduced dose bath to the rectum and the accurate IGRT techniques may be the explanation of the finding above. Another result of our trial is that treatment side effects did not correlate with IGRT regimens (fiducial, kV CBCT or MV CBCT).

At 30-month follow-up time we experienced excellent biochemical control (98%).

There are several limitations of our study. One is the relatively short follow-up time. Longer follow-up is necessary to evaluate long-term toxicity and biochemical control. Another limitation is the lack of patient-reported toxicity,therefore we may underestimate the toxicity rates. Furthermore, this study was not a randomized one. We did not compareour SIB treatment protocol to a conventionally fractionated treatment schedule.

## Conclusion

WPRT with SIB tothe prostate, seminal vesicles or positive pelvic lymph nodes is a feasible and safe technique for patientswith intermediate and high risk localized, locally advanced and node positiveprostate cancer. According to our results this treatment seems to be associated with a tolerable frequency and severity of acute GU and GI toxicities. The rate of severe late GI and UG toxicities are low and comparable to rates with conventionally fractionated treatments. This technique provides shorter overall treatment time compared to conventional fractionation thus sparing treatment capacity on the linear accelerator. Additional validation with a longer follow-up is needed.
